# Supplementary material for: Route learning and transport of resources during colony relocation in Australian desert ants
Source: Learn Behav. 2024 Oct 22;53(2):204–16. doi: 10.3758/s13420-024-00652-1 (PMC12092541; doi:10.3758/s13420-024-00652-1)
Supplement: Supplementary file 1 — Supplementary file1 (DOCX 1586 KB) [file 13420_2024_652_MOESM1_ESM.docx]

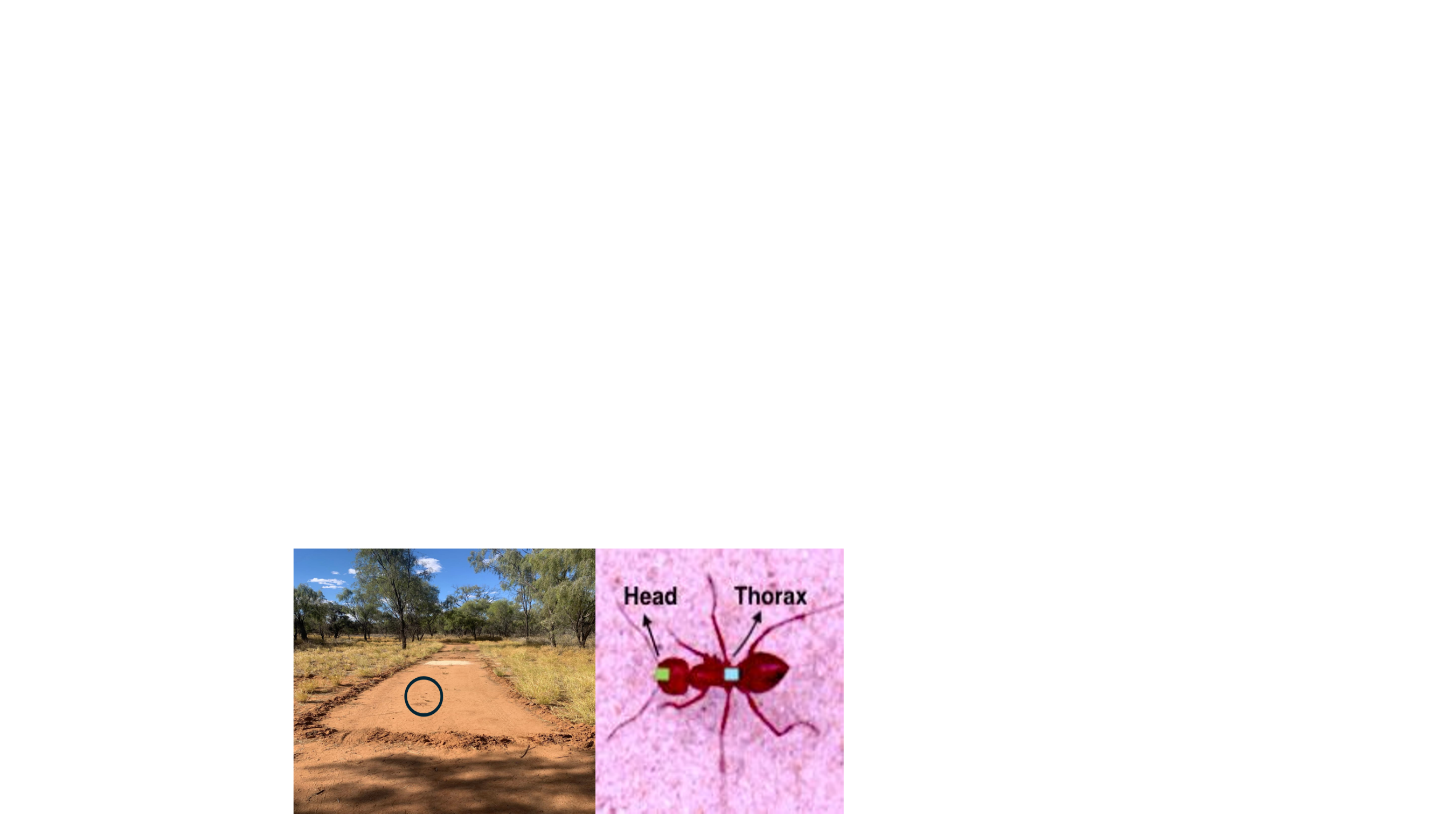


S. Figure 1. The left image shows the nest habitat of red honey ant. The black circle denotes the position of the nest. The right image shows the body positions used to annotate ants on video records: Front of the head and Mid thorax.


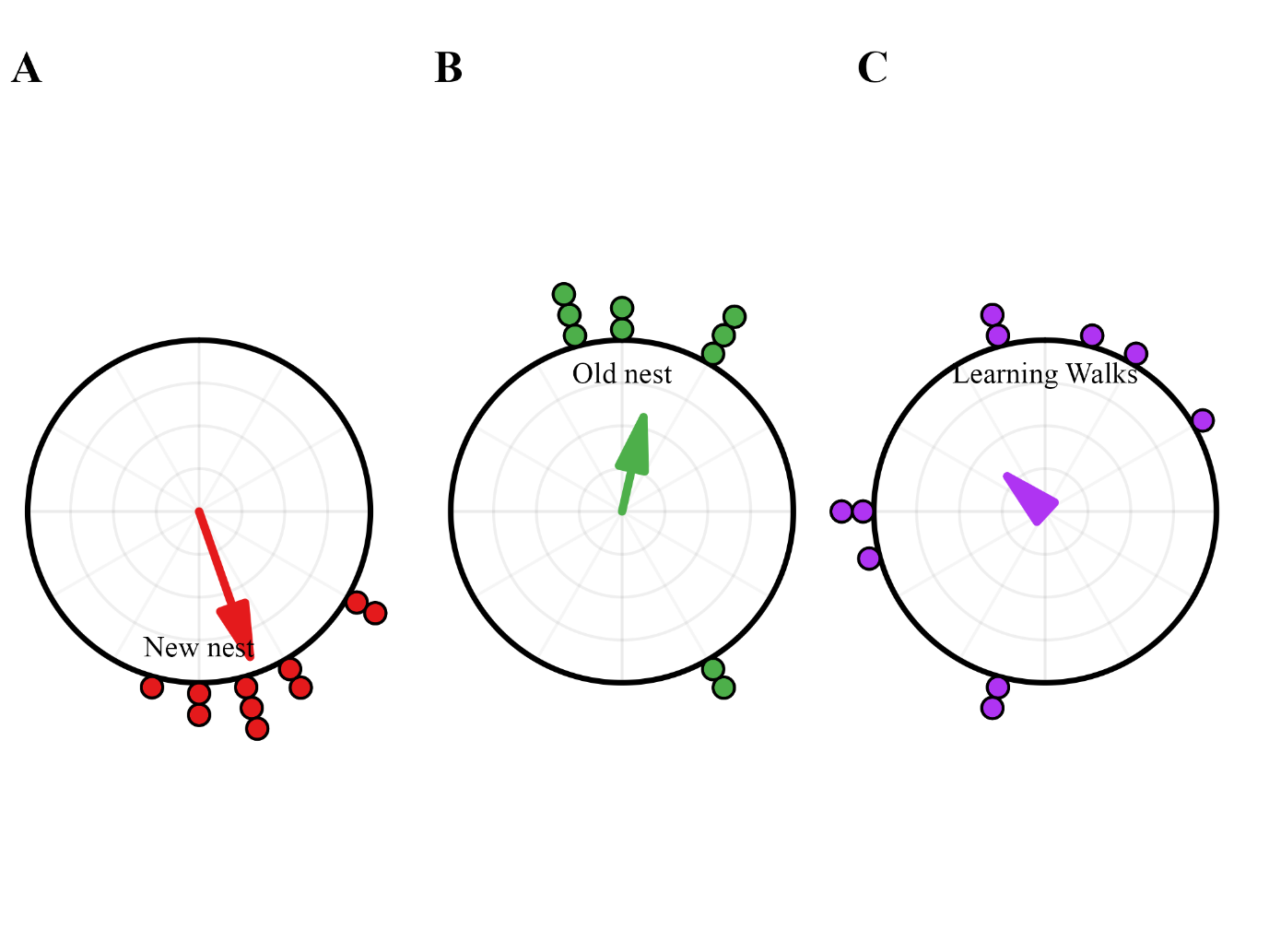


S. Figure 2. Circular histograms show the centroid direction of the ants during the relearning walk at Old nest (A), relearning walks at the New nest (B) and naive learning walks. In the histograms, the nest direction is set at 0°. The arrows denote the length and direction of the mean vector. The Rayleigh uniformity test and the distribution of headings with the *V* test showed significance in centroid heading toward the New nest at Old nest relearning walks (Z = 9.16, *p* ≤ 0.001; *V* test: Z = 4.72, *p* ≤ 0.001), whereas the New nest relearning walks (Z = 1.16, *p* = 0. 076; *V* test: Z = 1.02, *p* = 0.81) and learning walks (Z = 0.16, *p* = 0.41; *V* test: Z = 0.74, *p* = 0.24) showed random distribution in their centroid direction.
